# Evaluation and Characterization of Hard-Shell Capsules Formulated by Using Goatskin Gelatin

**DOI:** 10.3390/polym14204416

**Published:** 2022-10-19

**Authors:** Zilhadia Zilhadia, Yahdiana Harahap, Irwandi Jaswir, Effionora Anwar

**Affiliations:** 1 Department of Pharmacy, Faculty of Health Sciences, Universitas Islam Negeri Syarif Hidayatullah Jakarta, Banten 15412, Indonesia; 2 Faculty of Pharmacy, Universitas Indonesia, Depok 16424, Indonesia; 3 Faculty of Military Pharmacy, Republic of Indonesia Defense University, Bogor 16111, Indonesia; 4 International Institute for Halal Research and Training, International Islamic University Malaysia, Kuala Lumpur 53100, Malaysia; 5 Faculty of Pharmacy, Universitas Ahmad Dahlan, Yogjakarta 55000, Indonesia

**Keywords:** gelatin, goatskin, hard-shell capsule

## Abstract

Gelatin is used as an additive in medicine, food, and cosmetics. Gelatin from goatskin is a new excipient that has not been explored by researchers, including for hard-shell capsules. The aim of this study was to evaluate and characterize the hard-shell capsules produced from goatskin gelatin. The goatskin gelatin was extracted by an acid hydrolysis method, and the functional properties were investigated. Hard-shell capsules were then produced from goatskin gelatin, evaluated, and characterized. The gelatin extracted from goatskin had 56.9% ± 0.95 clarity and a pH of 5.11 ± 0.09, 97.51% ± 1.1 protein content, 9.23% ± 0.08 water content, 0.18% ± 0.07 ash content, 2.08% ± 0.35 fat content, gel strength of 298 ± 2.64 gbloom, and viscosity of 27.33 ± 2.07 mPs. The gelatin has met the requirements to be made into hard-shell capsules. The average weight of the hard-shell capsules produced was 96.9 mg with 8.69 standard deviation. The average size of the body and cap length was 18.84 ± 0.64 mm and 10.98 ± 0.30 mm, respectively. The results of capsule evaluation and characterization were as follows: the pH was 4.82 ± 1,27, water content was 10.03 ± 0.21, disintegration time was 4.02 ± 2.09 min, and there was no microbial growth. Thus, the capsules made have met the requirements and can be produced in a large quantity.

## 1. Introduction

Empty capsules (hard-shell capsules) are one of the oldest pharmaceutical dosage forms, which has been used since the Ancient Egyptian era. Hard-shell capsules are used for drugs, vitamins, minerals, and nutraceutical preparations because they can mask the unpleasant taste and odor of drugs. The hard capsule shell protects the drug inside from extreme external influences, such as light, oxygen, contamination, and microbial growth [1,2]. Although it has been a long time, the use of hard-shell capsules as one of the dosage forms continues to increase. The global empty capsule market was valued at $2382.7 million in 2020 and is projected to reach $5230.4 million by 2030, registering a CAGR of 8.1% from 2021 to 2030 [3].

The most popular empty capsules on the market are gelatin capsules. They are named gelatin capsules because in the formula the largest component after water is gelatin which ranges from 30% to 45% [4]. Gelatin is a solid colloidal system (protein) in a liquid (water), which at high temperatures and high water-content has a liquid ability called the sol or hydrosol phase. On the other hand, at a low temperature and low water-content, gelatin has a coarser and more concentrated structure called the gel phase. In general, sol/gel transitions can be evaluated within a temperature range of about 5–60 °C, and the gelatin concentration can vary between 0.5 and 50%, depending on the quality of the gelatin. Heating and adding water will convert gelatin into a sol phase, whereas cooling and reducing water will convert gelatin into a gel phase [2,5,6].

The most common sources of gelatin for industrial production are porcine and bovine skins and the bones of porcine animals and cattle [7]. Globally, more than 45% of gelatin comes from porcine. This source of gelatin is a major concern for Muslim and Jewish consumers because porcine gelatin is not permitted (nonhalal and nonkosher) for consumption [8,9]. Ninety-five percent of global gelatin production is sourced from animals that are not halal-friendly nor kosher-friendly [10]. Halal gelatin and kosher gelatin are important in the pharmaceutical and nutraceutical sectors in emerging markets, such as the Middle East and Asia, which have large Muslim populations and substantial growth rates. The world’s Muslim population is predicted to increase and reach 23.4% of the total population of the world. With the increase in the global Muslim population, the global demand for halal capsule shells will continue to increase [11,12]. Therefore, alternative materials are needed as a source of halal gelatin which can be used in hard-shell capsule formulations.

Another halal and kosher friendly animal that has not been widely developed as a source of gelatin is goat. Goat meat is used by Indonesians as food and for Islamic ceremonies (ritual animal sacrifice called Qurban and Aqiqah). However, goatskin is often discarded by residents because it is less useful and has a low price. Discarded goatskin can become waste and damage the environment. The proper use of goatskin can increase its economic value and reduce environmental problems. Goatskin is a resource that should be utilized as much as possible, including as a potential source of gelatin [13,14].

Zilhadia et al. (2018) have developed gelatin extracted from goatskin. Based on the characteristic test, the goatskin gelatin has good properties to be used as an ingredient in the food, medicine, and cosmetic industries [5]. In this study, hard-shell capsules are formulated from goatskin gelatin, and the properties of the hard-shell capsules are evaluated.

## 2. Materials and Methods

### 2.1. Materials

The raw material was goatskin purchased from a slaughterhouse in Depok, Indonesia. The materials for gelatin extraction were sodium sulfide (VWRChemicals, Leuven, Belgium), calcium hydroxide, sodium hydroxide, hydrochloride acid, and distilled water purchased from Sigma-Aldrich, St. Louis, MO, USA. The materials for formulating the capsules were methyl paraben (Ueno Fine Chemical Industry, Osaka, Japan), propylparaben (Ueno Fine Chemical Industry, Osaka, Japan), titanium dioxide (Nanjing Bersilion Technology, Nanjing, China), sodium laurel sulfate (Anmol Chemical, Mumbai, India). The material for determination of properties gelatin were nutrient agar (Oxoid, London, UK) and potato dextrose agar (Oxoid, London, UK), alpha-aminobutyric acid (Sigma-Aldrich, St. Louis, MO, USA), and Fluor borate (Sigma-Aldrich, St. Louis, MO, USA).

### 2.2. Gelatin Extraction

The extraction procedure followed the gelatin extraction method by Zilhadia et al. (2018) with slight modifications [5]. The goatskin from slaughterhouse was cleaned, and the fat and meat were removed by using scissors. For the unhairing process, the goatskin was soaked in a mixture of sodium sulfide and calcium hydroxide. The hair of goatskin was removed and cleaned with tap water until the skin pH was neutral. The skin was then cut into 2–3 cm^2^ pieces using a scalpel and ready to use.

The goatskin was soaked in 4% (*v*/*v*) hydrochloric acid at 5 °C for 48 h. The acid-treated goatskin was then washed with distilled water until the washing water was clean. The final extraction was carried out in distilled water at 60 °C for 9 h. The extract obtained was filtered through a Buchner funnel with Whatman filter paper No.1. The filtrate was evaporated in an oven at 60 °C for 2 h. The filtrate was then cooled in the refrigerator at 5 °C until a liquid gelatin was formed. The liquid gelatin was dried using an oven at 60 °C for 20 h. The dried gelatin was then ground to produce powdered gelatin. The powdered gelatin was weighed to calculate the yield.

### 2.3. Clarity of Goatskin Gelatin

The clarity test of goatskin gelatin was conducted by using a spectrophotometer (Hitachi U-2910, Tokyo, Japan) to measure the % transmittance (%T) of gelatin solution [8,15]. The gelatin was weighed for 6.67 g and dissolved in distilled water at 60 °C for 1 h until the volume reached 100 mL. The %T of the gelatin solution was measured at 620 nm wavelength, and distilled water was used as the blank.

### 2.4. Measurement of Gelatin Solution pH

The pH measurement was carried out according to the GMIA method (2012). One gram of gelatin was dissolved in distilled water at a temperature of 45 °C with a volume of 100 mL. The solution was allowed to reach a room temperature [4,16]. The pH of the solution was then measured using a pH meter (Horiba, Kyoto, Japan).

### 2.5. Proximate Analysis

The moisture, ash, and fat contents of the goatskin gelatin were determined according to the AOAC (2000) methods number 927.05, 942.05, and 920.39 B, respectively. The crude protein content was determined by estimating its total nitrogen content by using the Kjeldahl method according to the AOAC (2000) method number 984.13 [17]. All measurements were performed in triplicate.

### 2.6. Measurement of Gel Strength

The gel strength of the goatskin gelatin was determined by using the GMIA, 2012 method [4]. The goatskin gelatin (6.67 g) was weighed and dissolved in distilled water at 60 °C for 30 min until the volume was 100 mL. The solution was then cooled in the refrigerator at 7 °C for 16–18 h. The gel strength was measured by using a texture analyzer (TA-XT2, Godalming, UK) with a load strength of 30 kg equipped with 1.27 cm diameter flat plunger. The diameter of the test sample was 3.8 cm, and it was 2.7 cm in height. The gel strength was measured as the strength required by the plunger to press the gel as thick as 4 mm at a speed of 0.5 mm/s.

### 2.7. Measurement of Viscosity

The viscosity of 6.67% goatskin gelatin solution was evaluated by using a rotational viscometer (Brookfield, WI, USA). The measurement conditions were as follows: the temperature was maintained at 27 °C, the spindle was number 1, and the sample volume was 250 mL [18].

### 2.8. Determination of Microbial Levels

The measurement of the microbial levels was carried out according to the 4th edition of Indonesian Pharmacopoeia (1995). One gram of gelatin was dissolved in 10 mL of distilled water and then diluted to obtain a concentration of 100 ppm. One µL of the solution was taken and spread onto a solid medium and then incubated for 24 h. After incubation, the number of colonies were calculated [18].

### 2.9. Determination of Amino Acid Composition

Prior to measurements, gelatin was hydrolyzed to obtain free amino acids. Gelatin samples (100 mg) were hydrolyzed in an oven at 110 °C for 22 h. Hydrolyzed gelatin was added to 40 μL alpha-aminobutyric acid in hydrochloride acid and 460 μL distilled water. The solution (10 μL) was added to 70 μL AccQ Fluor borate and heated at 55 °C for 10 min, then cooled to room temperature. The sample was injected into the High-Performance Liquid Chromatography (Waters, Milford, MA, USA), detector PDA, flow rate 0.7 mL/min, eluent AccQTag Eluant A- HPLC grade 60% acetonitrile with gradient system, temperature 49 °C, column Waters AccQ Tag Ultra C18, 4 μm (3.9 × 150 mm) [5,8].

### 2.10. Determination of Microscopic Structure

Microscopic structure of goatskin gelatin was obtained using Scanning Electron Microscope (Quanta Inspect F50, Eindhoven, The Netherlands) equipped with field emission gun with 1.2 nm resolution and an energy dispersive X-ray spectrometer with an MnK resolution of 133 eV. The sample was dried then coated with gold for one hour on a coating machine. The sample was inserted into a SEM, and the morphology of the gelatin was observed [19].

### 2.11. Formulation of Hard-Shell Capsules from Goatskin Gelatin

The hard-shell capsules were prepared by using the following formula: 35% Gelatin, 0.3% titanium dioxide, 0.2% methyl paraben, 0.05% propyl paraben, 1% coloring agent, 0.1% sodium lauryl sulfate, and distilled water to obtain 100 mL solution. The gelatin concentration obtained was based on the optimization process (data not shown).

The goatskin gelatin was dissolved in purified water at 45 °C until it was completely dissolved. In another container, titanium dioxide, methyl paraben, propyl paraben, and sodium lauryl sulfate were dissolved in purified water at 45 °C. The obtained solution was then mixed with the gelatin solution at the same temperature until homogeny was reached. After mixing, the gelatin solution was poured into a mold pin at 45 °C. The hard gelatin capsules were manufactured by dipping stainless steel mold pins into a gelatin solution, drying the mold, stripping the capsules from the pins into a collate, trimming the caps and bodies, and joining the caps and bodies together [4,20].

### 2.12. Evaluation of the Hard-Shell Capsule Weight Variations

The determination of the capsule weight variations was performed according to the US pharmacopoeia 29 and National Formulary 24. Low variations in the capsule weight were negligible and could be accepted. Twenty (20) capsules were randomly taken, and their weight and average weight were determined. They met the requirement if each of the weights was within the limits of 90% and 110% of the average weight [21].

### 2.13. Evaluation of the Size Uniformity (Capsule Body and Cap)

The size uniformity test was carried out on 20 capsule shells. The capsule body length and capsule cap were measured one by one using a micrometer screw. The average body length and capsule cap were then calculated [22,23].

### 2.14. Moisture Content Test

The moisture content was measured based on the AOAC, 1995 method number 927.5. The container used was a sealed glass that had been dried until the weight was constant. One gram of capsules was placed in the container and put in an oven at 100 °C. The container and gelatin were cooled in a desiccator. This process was carried out until the weight was constant. The reduction in weight was calculated as a percentage of moisture content [24].

### 2.15. Disintegration Time Test

The disintegration time test was carried out by following the established methodology for European Pharmacopoeia 5, 2003 using a disintegration time tester (Erweka ZT 322, Langen, Germany). A basket-rack assembly was filled with 600 mL 0.1 N hydrochloride acid. Capsules and discs were placed in each of the 6 tubes of the basket. The device was connected to an electric current, and the temperature of the device was set at 37 °C. The equipment was operated until there was no residue in the filter. All of the six capsules had to disintegrate [25].

### 2.16. Measurement of the pH

One gram of capsules was put into 10 mL of distilled water until they were completely dissolved, and then the pH was measured with a pH meter (Horiba, Kyoto, Japan). The pH requirement for capsules is 4.5–5.5 [4].

### 2.17. Microbial Assay

This test was performed to check the presence of microbes, especially the total viable aerobic count in the capsule shells. One mL of capsule solution in water (100 ppm) was taken and spread into a solid medium. The medium that contained the capsule solution was incubated at 35–37 °C for 24–120 h. After incubation, the number of living colonies was calculated. The requirement is lower than 100 cfu/g for aerobic bacteria [4,18].

## 3. Results and Discussion

### 3.1. Yield of Gelatin

The yield of extracted goatskin gelatin was 12.74% ± 0.87 (wet weight basis). The yield of goatskin gelatin in this study was greater than that of Zilhadia et al., 2018 (10.26% ± 1.07) *p* < 0.05. The yield increased when the hydrochloric acid concentration was raised to 4%. A higher yield indicates enough time to process the bond breaking in collagen. The ionic strength of the solution at an acidic pH facilitates the swelling process caused by the repulsion on the structure of collagen. With the loss of bonding power, warm water will be able to penetrate effectively into the matrix [5,26].

**Figure 1:**
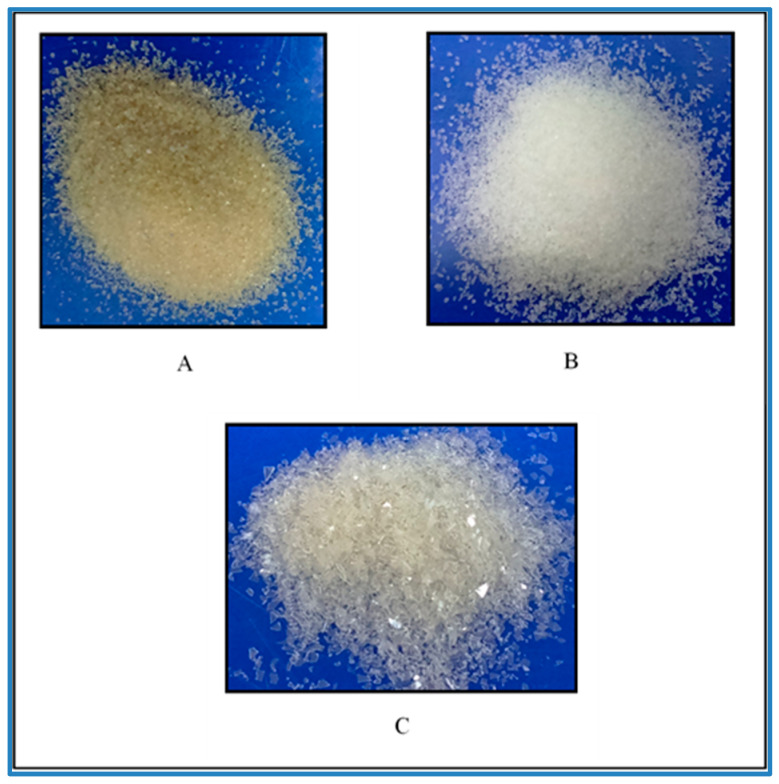
Gelatin extracted from goatskin compared to porcine gelatin and bovine gelatin. (**A**) Bovine gelatin; (**B**) porcine gelatin; (**C**) goatskin gelatin.

Organoleptic properties are the physical properties of a substance which can be seen by unaided eyes, so these properties become the first evaluation of a substance. Gelatin occurs from a light amber to a faintly yellow-colored, vitreous, and brittle solid. It is practically odorless and tasteless and is available as translucent sheets, flakes, and granules, or as coarse powder [27]. The goatskin gelatin obtained was yellowish white in the form of coarse powder, and the powder size was less uniform (Figure 1). The goatskin gelatin was yellower than porcine gelatin (Sigma, St. Louis, MO, USA) but whiter compared to bovine gelatin (Sigma, St. Louis, MO, USA).

### 3.2. Clarity of Gelatin Solution

The clarity of gelatin solution (in water) is an important indicator to be applied in the drug, food, and cosmetic fields. It is directly related to its aesthetic value and the color produced. Gelatin color is influenced by the extraction methods and raw materials used [28]. In this study, the clarity determination was carried out by comparing water transmittance (the value of water transmittance is 100) with the gelatin solution transmittance as measured by a spectrophotometry. The transmittance value of the gelatin obtained was 56.9% ± 0.95.

The clarity value of goatskin gelatin is low. It can be caused by inorganic contaminants, proteins, and mucosal compounds which are inseparable during extraction. Inorganic contaminants can be insoluble particles that spread light and particles that cause turbidity in the solution. In addition, the value of clarity is strongly influenced by the filtration process [8]. Although the gelatin transmittance is 56.9%, it physically looks like a clear solution, making it possible to be used in the production of hard-shell capsules.

### 3.3. pH

Gelatin that meets the requirement has a pH of 3.8–5.5 [4]. A pH has an effect on the properties of gelatin as an excipient ingredient. The pH of gelatin is related to the process or treatment. A pH of 5 gives an ideal effect on the gel strength [8]. In this study, the pH of goatskin gelatin was 5.11 ± 0.09, indicating that it has fulfilled the requirement. 

### 3.4. Proximate Composition

The proximate compositions of goatskin gelatin consist of protein, moisture, ash, and fat contents. The main content of gelatin is protein [29,30,31]. High levels of protein indicate good quality gelatin. The protein content in the goatskin gelatin was 97.51% ± 1.1, which shows that the main component of the composition of goatskin gelatin is protein.

Moisture content becomes the test required for gelatin. Water in a substance is one of the factors that influence metabolic activities, such as enzyme activity, microbial activity, and chemical activity. Meanwhile, water in gelatin determines stability, acceptability, freshness, texture, taste, and durability of food and drugs. In addition, dry gelatin is stable in the air. The moisture content of gelatin is between 8–13% [4], and the moisture content of the goatskin gelatin in this study was 9.23% ± 0.08.

Ash content is one of the requirements that must be fulfilled by gelatin. Low ash levels indicate good quality gelatin [30,31]. The requirement for gelatin ash levels is not more than 2–2.5% [27,32]. The ash content of the goatskin gelatin obtained was 0.18% ± 0.07.

Fat content is one parameter that indicates the purity of gelatin. The lower the fat content, the purer the gelatin produced [31]. In this study, the fat content of the goatskin gelatin was 2.08% ± 0.35. According to the requirement, the fat content should be <5% [2].

The presence of protein at very high levels and ash, lipid, and moisture of goatskin gelatin at very low levels shows that the goatskin gelatin meets the requirements [31].

### 3.5. Gel Strength

Gel strength is one of the most important functional properties of gelatin [8,31]. The gel strength of the goatskin gelatin obtained was 298 ± 2.64 gbloom. Gel strength is categorized into three groups of low bloom (<150 gbloom), moderate bloom (150–220 gbloom), and high bloom (220–300 gbloom) [29]. Based on this category, the gel strength of the goatskin gelatin is classified as high gel strength. For the manufacture of capsule shells, the strength of good gelatin is 240–300 gbloom [4]. Therefore, based on the gel strength, the goatskin gelatin is suitable for the production of hard-shell capsules.

### 3.6. Viscosity

Viscosity is an important characteristic of gelatin included at the time of capsule shell making for both hard-shell capsules and soft capsules [4,33]. The viscosity of gelatin solution has a direct effect when a mold pin is dipped, lifted, and dried. If the viscosity of gelatin solution is not suitable, thick capsules with ununiform weight and size are produced. The viscosity of the goatskin gelatin was 27.33 ± 2.07 mPs. According to GMIA, the viscosity of capsule shells is 25–55 mPs [4]. Therefore, the viscosity of the goatskin gelatin solution can be used to prepare capsule shells.

### 3.7. Microbial Assay

According to the United States Pharmacopeia 34 and National Formulary, pharmaceutical gelatin should not contain *Salmonella species* and *Escherichia coli* [34]. On the other hand, gelatin is stable in the air when it is dry but subject to microbial decomposition when it becomes moist [23]. Therefore, microbial assay is crucial in gelatin characterization. The results of microbial assay show that the gelatin does not contain Salmonella species and *E. coli*. There is no microbial growth because the final production stage is heating. After gelatin is produced, it is stored in airtight containers to protect it from the influence of air humidity.

### 3.8. Amino Acid Composition

The functional properties of gelatin are influenced by the amino acid composition of gelatin constituents. The amino acid composition of gelatin from goat skin, bovine skin, and porcine skin are shown in Table 1. 

The major amino acid composition of goatskin gelatin is glycine (29.13%), followed by proline (13.38%), arginine (10.03%), glutamic acid (9.60%), and alanine (8.43%). The major composition of bovine gelatin is glycine (31.15%), glutamic acid (12.31%), arginine (9.64%) alanine (9.62%), and proline (8.57%). Meanwhile, the content of porcine gelatin is glycine (28.24%), proline (14.12%), glutamic acid (11,52%), arginine (9.40%), and alanine (9.02). Based on the glycine and proline content as the major gelatin components, the goatskin gelatin seemed to be similar to porcine gelatin and differed from bovine skin gelatin. The five main amino acids in goatskin in this study were the same as the amino acids in the goatskin gelatin produced through the alkaline hydrolysis method conducted by Mad-Ali et al., 2018, which include glycine, proline, alanine, glutamic acid, and arginine [5,14].

The minor components (less than 2%) of the goat skin gelatin extracted were isoleucine (1.40%), methionine (1.15%), tyrosine (1.11%), histidine (1.03%), and cystine (0.01%). Cystine and tryptophan are not commonly present in gelatin. However, cystine was detected in this study. Sarbon et al. (2013) report that cystine is detected at 0.16% in gelatin derived from chicken skin and at 0.47% in bovine skin gelatin [29]. Cystine is detected due to the presence of a few hairs left during the unhairing process [5].

### 3.9. Microscopic Structure of Goatskin Gelatin

**Figure 2:**
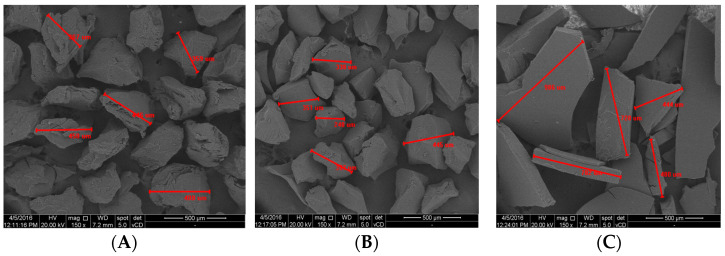
Microscopic structure of goatskin gelatin compared to porcine gelatin and bovine gelatin. (**A**) bovine gelatin; (**B**) porcine gelatin; (**C**) goatskin gelatin.

Microscopic structure of goatskin gelatin compared to bovine gelatin and porcine gelatin can be seen in Figure 2. 

Bovine gelatin has an irregular, asymmetrical shape, but the particles look more uniform with a length between 359 μm and 488 Bovine gelatin has an irregular, asymmetrical shape, but the particles look more uniform with a length between 359 μm and 488 μm. The surface has pores and looks rougher. Porcine gelatin has an irregular, asymmetrical shape and a non-uniform size with a particle length between 248 μm and 445 μm. The surface is non-porous, and among the three gelatin types, pork gelatin has the smallest particles. Meanwhile, goatskin gelatin is flat, with an extremely variable size ranging from 444 μm to 986 μm. The surface is smooth, non-porous, but irregular in shape.

The surface morphology of goatskin gelatin is different from that of bovine gelatin and porcine gelatin. Bovine and porcine gelatin have a round and more regular shape while goatskin gelatin is flat and less uniform. This is due to differences in the refining process. The gelatin industry reduces the size of gelatin using machines. Meanwhile, the goatskin gelatin in this study was refined using a blender. Blender blades produce non-uniform shapes. The surface has pores and looks rougher. Porcine gelatin has an irregular, asymmetrical shape and a non-uniform size with a particle length between 248 μm and 445 μm. The surface is non-porous, and among the three gelatin types, pork gelatin has the smallest particles. Meanwhile, goatskin gelatin is flat, with an extremely variable size ranging from 444 μm to 986 μm. The surface is smooth, non-porous, but irregular in shape. 

The surface morphology of goatskin gelatin is different from that of bovine gelatin and porcine gelatin. Bovine and porcine gelatin have a round and more regular shape while goatskin gelatin is flat and less uniform. This is due to differences in the refining process. The gelatin industry reduces the size of gelatin using machines. Meanwhile, the goatskin gelatin in this study was refined using a blender. Blender blades produce non-uniform shapes.

### 3.10. Hard-Shell Capsule Formulation

Based on the optimization process, this study used gelatin with a concentration of 35%. The side of the hard-shell capsules was badly formed or too thin when the concentration of gelatin was about 30%. Meanwhile, when approximately 40% concentration was used, the hard-shell capsules were too thick.

The pharmaceutical industry requires commercial capsules that have attractive colors and are not transparent, so the drug powder inside the capsules is not visible. For this purpose, capsule shell formulas require an opaque agent in combination with a coloring agent. The most popular opaque agent is titanium dioxide [23]. Titanium dioxide is an inner and safe material and has been used in many applications for decades [35,36]. As for the opaque agent in this research, titanium dioxide was used.

Preservatives are one component that must be present in a capsule formula. Preservatives should meet two requirements, including fulfilling the legal aspect for the pharmaceutical industry and having a suitable curing effect on capsules. The capsule preservative used in this study was a combination of methyl paraben and propyl paraben with a bacteriostatic effect. The use of this combination is necessary since methyl paraben and propyl paraben are effective against microbes with a spectrum for different organisms [20].

Meanwhile, coloring is needed for several purposes. First, it is used to provide an aesthetic value for the capsules produced. Second, coloring functions to protect capsule contents from the influence of light, particularly for substances that are unstable when exposed to light. Third, coloring can be used as an identity of a capsule. Fourth, coloring also has a psychological role for patients to increase the curative value of drugs in capsules [2,20].

Then, sodium lauryl sulfate is used as a surfactant specifically mentioned in the American Pharmacopoeia as a processing aid in capsule production. With the presence of sodium lauryl sulfate, capsule shells can be separated from the mold after the drying process [20].

**Figure 3:**
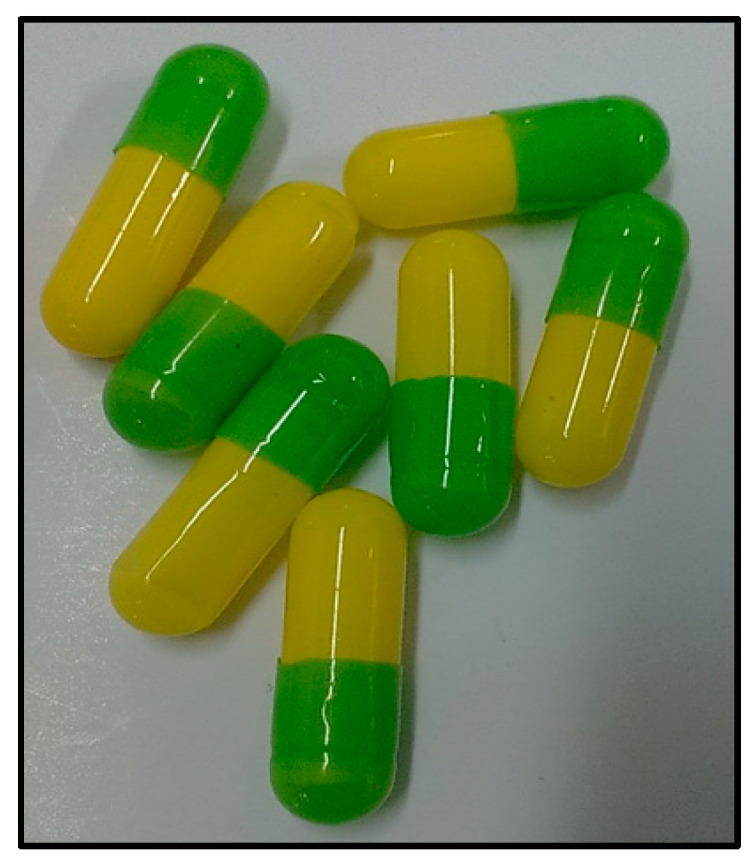
Hard-shell capsules produced from goatskin gelatin.

In the manufacturing process, the speed of metal molds in dipping and the viscosity of gelatin solution will affect the size and thickness of capsule shells. If the viscosity is too high and the metal molds are immersed for too long, a thick capsule shell will be produced. However, if the viscosity is too low and the dipping of the metal molds into the gelatin solution is too short, the capsule becomes thinner and brittle. A uniform capsule shell size is obtained from suitable viscosity. The capsule shells from the goatskin gelatin produced can be seen in Figure 3.

### 3.11. Uniformity of Weight and Size (Body and Cap Length)

The uniformity test of the weight and size of the hard-shell capsules produced from goatskin gelatin can be seen in Table 2. The requirement for the weight of hard-shell capsules sized 0 is 87–105 mg with a standard deviation of 6% [37,38]. Meanwhile, the average weight of the hard-shell capsules produced was 96.9 with 8.69% standard deviation. The obtained weight has met the requirement, but the standard deviation exceeds 6% because the production of the hard-shell capsules was performed manually.

The length requirement for the body and cap of capsules sized 0 is 17.9–18.9 mm and 10.20–11.00 mm, respectively [37,38]. Meanwhile, the average size of the body and cap length obtained was 18.84 ± 0.64 mm and 10.98 ± 0.30 mm. The length of the body and cap is within the range that meets the requirement. The uniformity of the weight and length of capsule shells is an important factor to ensure that the capsule shells are accepted by the medicine industry. The uniformity of weight and size affects the uniformity of content, which is directly related to the dose of drugs. Capsule uniformity increases productivity and production efficiency. Capsule shells with a larger or smaller size will be wasted, leading to an increase in the financing of capsule production [23].

### 3.12. Moisture Content of Capsules

A moisture test was carried out to evaluate the drying process of hard-shell capsules during production. High levels of water cause hard-shell capsules to quickly become muddy, making it difficult for storage and transportation. In addition, high water content of capsule shells is suitable for growing microbes. Low water levels cause capsule shells to be brittle and break easily. The requirement for the moisture content of hard-shell capsules is 13–16% [4,22]. Meanwhile, the moisture content of the hard-shell capsules produced was 10.03%, which shows that these capsules have fulfilled the requirement.

### 3.13. Disintegration

The purpose of the disintegration time test of hard-shell capsules in an acidic solution is to give an indication of the time required by the capsules to dissolve in gastric fluid and to release the drug in a capsule form [39,40,41]. The result obtained was 4.02 ± 2.09 min, which has met the requirement of less than 5 min.

The disintegration time of the hard gelatin capsule shells tested by Hunter et al., 1980 was 9 min [42]. In addition, Marvolla et al. (1988) tested the disintegration time of hard gelatin capsule shells filled with ethyl cellulose and polyethylene glycol 1500. The disintegration times obtained were 9 min and 23 min, respectively [43]. Thus, the disintegration time of the capsule shells in this study is better than that of the previous studies that used different ingredients.

### 3.14. Determination of pH

The capsule pH is a requirement that must be determined, and its value must range from 4.5 to 5.5 [4]. The pH value of the capsule shell solution obtained was 4.82 while the pH value of goatskin gelatin was 5.11. This shows that there is a decrease in the pH of the gelatin solution after it becomes hard-shell capsules. This is because the gelatin has been mixed with other excipients. However, the pH of the capsule shell solution made from goatskin gelatin has met the requirement.

### 3.15. Microbial Measurement

All pharmacopoeia requires the measurement of the microbial content of hard-shell capsules. GMIA (2012) provides specifications for the requirements of microbial content in hard-shell capsules as shown in Table 3. To perform the microbial growth test, the hard-shell capsule solution was spread into a solid medium and incubated for 24–48 h. After incubation, no microbial growth was seen. For the fungal growth test, the incubation was carried out for 72–120 h. The incubation results also showed that the gelatin was not overgrown by fungi. These results indicate that the preservative used (a combination of methyl paraben and propyl paraben) is effective in inhibiting microbial growth in the hard-shell capsules.

## 4. Conclusions

The percentage of the gelatin extracted from goatskin by using acid hydrolysis method was 12.74% ± 0.87. The physicochemical properties of the goatskin gelatin including the pH, moisture, ash, fat, protein content, viscosity, and microbial assay have met requirements. The amino acid composition of the goatskin gelatin has a similarity with that of porcine gelatin, with the main components being glycine, proline, arginine, glutamic acid, and alanine. The gel strength was 298 ± 2.64 gbloom, which is classified as high gel strength. The clarity of the gelatin was 56.9% ± 0.95. Although the clarity value was low, the gelatin solution physically looked clear. Therefore, goatskin gelatin has good characteristics and meets the requirements to be used as the main ingredient of hard-shell capsules.

The characterization and evaluation of the hard-shell capsules were carried out to determine whether the capsule shells met the pharmaceutical requirements. The average weight uniformity was appropriate, but the standard deviation was 8.69%, exceeding the requirement that should be 6%. The size uniformity, disintegration time, moisture content, pH, and microbial content have fulfilled the requirements of pharmaceutical capsules. Therefore, goatskin gelatin can be used as an ingredient in hard-shell capsule preparation and scaled up to produce hard-shell capsules in larger quantities.

## 5. Patents

Hard shell capsule produced from goatskin gelatin with formula that mention in this manuscript already registered as a patent with number IDP 000071325.

## Figures and Tables

**Table 1:** Amino acid composition of goatskin gelatin, porcine gelatin, and bovine gelatin.

Amino Acid	Goatskin Gelatin	Porcine Gelatin	Bovine Gelatin *
Arginine	10.03	9.40	9.64
Serine	3.95	3.61	4.14
Aspartic acid	4.54	5.65	5.99
Threonine	3.04	2.44	2.39
Glycine	29.13	28.24	31.15
Isoleucine	1.40	1.29	1.68
Proline	13.38	14.12	8.57
Alanine	8.43	9.02	9.62
Lysine	3.66	5.03	4.79
Glutamic acid	9.60	11.52	12.31
Valine	2.37	2.67	2.54
Phenylalanine	3.74	2.24	2.22
Histidine	1.03	0	0
Leusine	3.44	2.94	3.11
Tyrosine	1.11	0.53	0.38
Methionine	1.15	1.29	1.46
Cystine	0.01	0	0

* Source: Zilhadia et al., 2018 [5].

**Table 2:** Weight and size of hard-shell capsules produced by goatskin gelatin.

No	Weight (mg)	Length of Capsule (mm)	
		Body	Cap
1	99	18.7	11.13
2	103	18.73	10.62
3	107	18.55	10.98
4	96	18.78	10.87
5	95	18.88	10.98
6	83	18.87	10.63
7	84	20.21	11.11
8	87	18.06	11.3
9	89	18.18	11.3
10	99	18.84	11.06
11	85	18.23	10.24
12	100	18.59	11.15
13	99	19.73	11.05
14	115	18.15	10.76
15	102	19.01	10.86
16	104	19.07	11.31
17	88	20.35	11.45
18	98	19.11	11.09
19	97	18.21	11.17
20	108	18.48	10.6
Avarage	96.9	18.8365	10.98
Standard Deviation	8.69	0.64	0.30

**Table 3:** Microbial measurement of hard-shell capsules.

Microbes	Microbes of Hard Shell Capsules	Requirements *
*Escherichia coli*	Negative	Negative
*Salmonella*	Negative	Negative
Yeasts & Molds	Negative	Max 1000 cfu/g

* Source: Gelatin Handbook, 2019 [4].

## Data Availability

Not applicable.

## References

[1] Lachman L., Lieberman H.A., Kanig J.L. (1994). The Theory and Practice of Industry Pharmacy.

[2] Schrieber R., Herbert G. (2007). Gelatine Handbook: Theory and Industrial Practice.

[3] Empty Capsules Market by Type (Gelatin—Porcine, Bovine, Bone Meal), (HPMC, Pullulan), Functionality (Sustained-Release, Delayed-Release), Application (Antibiotics, Vitamins, Antacids), End User (Pharma, Nutraceuticals, Cosmetics) Forecast to 2023. https://www.marketsandmarkets.com/Market-Reports/empty-capsules-market-218018190.html?gclid=Cj0KCQjw_ez2BRCyARIsAJfg-kvnZnUDCgWcByEdOcdwEkkImBN2Hu-Y6Dq2CsHiLt5hWjpABZf61RMaAnC6EALw_wcB.

[4] Gelatin Manufacturers Institute of America (2019). Gelatin Handbook.

[5] Zilhadia, Yahdiana H., Irwandi J., Effionora A. (2018). Characterization and functional properties of gelatin extracted from goatskin. Int. Food R. J..

[6] Karim A.A., Bhat R. (2008). Gelatin alternatives for the food industry: Recent developments, challenges and prospects. Trends Food Sci. Technol..

[7] Derkach S.R., Kolotova D.S., Kuchina Y.A., Shumskaya N.V. (2022). Characterization of Fish Gelatin Obtained from Atlantic Cod Skin Using Enzymatic Treatment. Polymers.

[8] Shyni K., Hema G.S., Ninan G., Mathew S., Joshy C.G., Lakshmanan P.T. (2014). Isolation and characterization of gelatin from the skins of skipjack tuna (*Katsuwonus pelamis*), dog shark (*Scoliodon sorrakowah*), and rohu (*Labeo rohita*). Food Hydrocoll..

[9] Venien A., Levieux D. (2005). Differentiation of bovine from porcine gelatines using polyclonal anti-peptide antibodies in indirect and competitive indirect ELISA. J. Pharm. Biomed. Anal..

[10] Zin Z.M., Sarbon N.M., Zainol M.K., Jaafar N.A., Syukri M.M., Rahman A.H. (2020). Halal and Non-Halal Gelatine as a Potential Animal By-Products in Food Systems: Prospects and Challenges for Muslim Community. Proceedings of the First International Conference on Science, Technology, Engineering and Industrial Revolution.

[11] Rakhmanova A., Khan Z.A., Sharif R., Xin L. (2018). Meeting the requirements of halal gelatin: A mini review. Food Process. Technol..

[12] Irwandi J., Faridayanti S., Mohamed E.S.M., Hamzah M.S., Torla H.H., Che Man Y.B. (2009). Extraction and characterization of gelatin from different marine fish species in Malaysia. Int. Food Res. J..

[13] Jayathilakan K., Sultana K., Radhakrishna K., Bawa A.S. (2012). Utilization of byproducts and waste materials from meat, poultry and fish processing industries: A review. J. Food Sci. Technol..

[14] Mad-Ali S., Benjakul S., Prodpran T., Maqsood S. (2016). Characteristics and Gel Properties of Gelatin from Goat Skin as Influenced by Alkaline-pretreatment Conditions. Asian Australas. J. Anim. Sci..

[15] Avena-Bustillos R.J., Olsen C.W., Olsun D.A., Chiou B., Yee E., Bechtel P.J., Hugh T.H. (2006). Water Vapor Permeability of Mammalian and Fish Gelatin Films. Food Eng. Phys. Prop..

[16] Jamilah B., Harvinder K.G. (2002). Properties of gelatins from skins of fish black tilapia (*Oreochromis mossambicus*) and red tilapia (*Oreochromis nilotica*). Food Chem..

[17] Association of Official Analytical Chemists (2000). Official Methods of Analysis.

[18] Ministry of Health Republic of Indonesia (2014). Indonesia Pharmacopoeia.

[19] Binulal N.S., Natarajan A., Menon D., Bhaskaran V.K., Mony U., Nair S.V. (2012). Gelatin nanoparticles loaded poly (ε-caprolactone) nanofibrous semi-synthetic scaffolds for bone tissue engineering. Biomed. Mater..

[20] Podczeck F., Jones B.E. (2004). Pharmaceutical Capsule.

[21] The United States Pharmacopeial Convention (2005). The United States Pharmacopeia 29 and National Formulary 24.

[22] Stegemann S., Bornem C. (2002). Hard Gelatin Capsules Today and Tomorrow.

[23] Allen L., Ansel H. (2014). Ansel’s Pharmaceutical Dosage Forms and Drug Delivery Systems.

[24] Association of Official Analytical Chemists (1995). Official Methods of Analysis.

[25] Directorate of the Council of the Europe (2022). European Pharmacopoeia.

[26] Ahmad M., Benjakul S. (2011). Characteristics of gelatin from the skin of unicorn leatherjacket (*Aluterus monoceros*) as influenced by acid pretreatment and extraction time. Food Hydrocoll..

[27] Rowe R.C., Sheskey P.J., Quinn M.E. (2009). Handbook of Pharmaceutical Excipients.

[28] Ockerman H.W., Hansen C.L. (1999). Glue and gelatin. Animal By-Product Processing and Utilization.

[29] Sarbon N.M., Badii F., Howell N. (2013). Preparation and characterisation of chicken skin gelatin as an alternative to mammalian gelatin. Food Hydrocoll..

[30] Balti R., Jridi M., Sila A., Souissi N., Nedjar-Arroume N., Guillochon D., Nasri M. (2011). Extraction and functional properties of gelatin from the skin of cuttlefish (Sepia officinalis) using smooth hound crude acid protease-aided process. Food Hydrocoll..

[31] Jellouli K., Balti R., Bougatef A., Hmidet N., Barkia A., Nasri M. (2011). Chemical composition and characteristics of skin gelatin from grey triggerfish (*Balistes capriscus*). Food Sci. Technol..

[32] Jones N.R., Ward A.G., Courts A. (1977). Uses of gelatin in edible products. The Science and Technology of Gelatin.

[33] Gudmundsson M., Hafsteinsson H. (1997). Gelatin from cod skins as affected by chemical treatments. J. Food Sci..

[34] The United States Pharmacopeial Convention (2011). The United States Pharmacopeia 34 and National Formulary 29.

[35] Skocaj M., Filipic M., Petkovic J., Novak S. (2011). Titanium dioxide in our everyday life; is it safe. Radiol. Oncol..

[36] Demina P.A., Grigoriev D.O., Kuz’micheva G.M. (2017). Preparation of pickering-emulsion-based capsules with shells composed of titanium dioxide nanoparticles and polyelectrolyte layers. Colloid J..

[37] Fauzi M.A.R.D., Pudjiastuti P., Wibowo A.C., Hendradi E. (2021). Preparation, Properties and Potential of Carrageenan-Based Hard Capsules for Replacing Gelatine: A Review. Polymers.

[38] Ridgway K. (1987). Hard Capsules: Development and Technology.

[39] Mahato R., Narang A. (2018). Pharmaceutical Dosage Forms and Drug Delivery.

[40] Carstensen J.T. (2001). Advanced Pharmaceutical Solids.

[41] Hillery A.M., Llyod A.W., Swarbrick (2005). Drug Delivery and Targetting for Pharmacist and Pharmaceutical Scientist.

[42] Hunter E., Felt J.T., Calvert R.T., Sharma H. (1980). In vivo disintegration of hard gelatin capsules in fasting and nonfasting subjects. Int. J. Pharm..

[43] Marvolla M., Hannula A., Westermack E., Happonen I., Kopra T. (1988). Disintegration of hard gelatin capsule formulations in the dog stomach-a radiological study. Int. J. Pharm..

